# Epidemiology of *Giardia intestinalis* in non-human primates and their caregivers: A study from Czech zoos

**DOI:** 10.1016/j.onehlt.2025.101176

**Published:** 2025-08-19

**Authors:** Kristýna Brožová, Anna Šejnohová, Monika Koutenská, Zuzana Pavlíčková, Milan Jirků, Eliška Zimmelová, Oldřiška Kadlecová, Klára J. Petrželková, Kateřina Jirků

**Affiliations:** a Institute of Parasitology, Biology Centre, the Czech Academy of Sciences, 370 05 České Budějovice, Czech Republic; b Faculty of Science, University of South Bohemia, 370 05 České Budějovice, Czech Republic; c Institute of Vertebrate Biology, Czech Academy of Sciences, Květná 8, 603 65 Brno, Czech Republic

**Keywords:** *Giardia intestinalis*, Intestinal protist, Non-human primates, Caregivers, Zoological gardens, Prevalence, Molecular diagnostics, Assemblage, Zoonotic transmission

## Abstract

*Giardia intestinalis* is a globally distributed protist whose epidemiology appears to be more complex than previously assumed. Recent studies suggest that it frequently asymptomatically colonizes the gastrointestinal tract of both humans and animals, yet its transmission dynamics remain poorly understood—particularly in shared environments involving close human–animal contact. Adopting a One Health perspective, this study investigated the occurrence of *G. intestinalis* in captive non-human primates (NHPs) and their caregivers across six zoological gardens in the Czech Republic, with a particular focus on the potential for zoonotic transmission. A total of 179 fecal samples (159 from 37 NHP species and 20 from caregivers) were analyzed using qPCR. *Giardia* was detected in 47 % of NHPs and 30 % of caregivers. Assemblage B was the most frequently identified genotype; however, genotyping was challenging in samples with low fecal protist load, limiting the ability to accurately assess transmission pathways. These findings reveal a high prevalence of *G. intestinalis* in zoo environments and underscore the importance of improved genotyping tools and further research into its transmission across species boundaries in human-managed settings.

## 1 Introduction

*Giardia intestinalis* is a globally distributed intestinal protist that infects both humans and animals, causing an estimated 180 million symptomatic cases annually [1]. Although traditionally associated with giardiasis, an acute diarrheal disease, recent research shows that *G. intestinalis* can also persist asymptomatically in the gastrointestinal tract of both healthy individuals and animals [2,3].

Genetically, *Giardia* represents a species complex comprising at least eight distinct assemblages (A–H) [2]. Assemblages A and B are most commonly found in humans and non-human primates (NHPs), and their broad host range underpins their zoonotic potential [4]. The remaining assemblages are typically host-specific to animals [5], although sporadic detections in humans have also been reported [6,7]. Growing evidence suggests that these assemblages may in fact represent distinct species [8].

In NHPs, *Giardia* has been detected in both wild and captive populations [[9], [10], [11], [12], [13], [14]], with prevalence in European zoological gardens ranging from 17 % to 70 % [9,11,[15], [16], [17], [18]]. In contrast, data on infections among zoo caregivers are scarce, with only a few studies reporting low prevalence (1.4–11 %) [15,19]. This limited evidence stands in sharp contrast to the frequent and close contact between NHPs and their caregivers, which creates conditions favorable for cross-species transmission. Zoonotic assemblages A and B have been found in both humans and NHPs, and phylogenetic evidence indicates that transmission can occur in both directions—zoonotic and anthroponotic [15,20]. However, the frequency, directionality, and underlying mechanisms of such events remain poorly understood.

A range of diagnostic methods is available for detecting *Giardia,* from traditional coproscopic techniques to modern molecular tools [21,22]. While flotation and sedimentation are still widely used, their sensitivity is limited due to intermittent cyst shedding [21]. Molecular approaches, including real-time PCR (qPCR) and conventional PCR (cPCR), offer higher sensitivity and allow for assemblage-level genotyping [23]. Nevertheless, their performance can be affected by low protist loads, PCR inhibitors (e.g., bile salts or polysaccharides), and the predominance of host or dietary DNA in fecal samples [21]. These limitations complicate the detection of low-intensity infections and reduce the reliability of assemblage identification, thus hindering accurate assessment of zoonotic transmission routes [6,24].

This study investigates the prevalence and assemblage distribution of *G. intestinalis* in captive NHPs and their caregivers across six zoological gardens in the Czech Republic. By focusing on a high-contact environment and zoonotic assemblages, it contributes to a One Health understanding of *Giardia* transmission dynamics at the human–NHP interface, with implications for both animal welfare and public health.

## 2 Materials and methods

### 2.1 Sample collection

Fecal samples from both non-human primates (NHPs) and their caregivers were collected between 2020 and 2022 in six zoological gardens in the Czech Republic (*Na Hrádečku, Hodonín, Děčín, Olomouc, Brno*, and *Dvůr Králové*). This study builds upon our previous publication Šejnohová et al. [25], in which we employed specific qPCR assays to detect the other intestinal protists including *Blastocystis* sp. and *Dientamoeba fragilis*. In this follow-up study, we focus specifically on the occurrence of *Giardia intestinalis* and also interpret its presence in the context of previously detected protists.

Fecal samples from NHPs, representing 37 species (Table 1), were collected by responsible caregivers from the floors of indoor enclosures during routine maintenance. Samples were collected individually, not pooled. Caregivers used sterile sampling kits consisting of a tube, spatula, and gloves. The number of samples collected from each zoo was as follows - 11 from *Na Hrádečku*, 5 from *Hodonín*, 16 from *Děčín*, 93 from *Olomouc*, 22 from *Brno*, and 12 from *Dvůr Králové*. Detailed information about housing conditions—such as the number of enclosures per zoo, the number of NHPs per enclosure, and whether caregivers rotated between enclosures during sampling—was not available. Similarly, data on individual NHPs' health status and antiparasitic treatment schedules were not provided, although routine antiparasitic treatment is administered in all participating zoos.

**Table 1:** An overview of the occurrence of *Giardia intestinalis* detected using qPCR in different non-human primate (NHP) species in Czech zoological gardens. The table categorizes each NHP species under its respective family and suborder, along with the total number of samples analyzed.

Host species	Family	Number of samples	Number of *Giardia* positive samples
*Ateles geoffroyi vellerosus*	Atelidae	4	2
*Callimico goeldii*	Callitrichidae	2	1
*Callithrix jacchus*	Callitrichidae	1	1
*Callithrix penicillata*	Callitrichidae	13	5
*Callithrix pygmaea*	Callitrichidae	2	1
*Cercopithecus campbelli*	Cercopithecidae	1	0
*Cercopithecus nictitans*	Cercopithecidae	1	1
*Colobus angolensis*	Cercopithecidae	2	1
*Erythrocebus patas*	Cercopithecidae	6	2
*Eulemur albifrons*	Lemuridae	6	0
*Eulemur macaco*	Lemuridae	11	4
*Eulemur rufifrons*	Lemuridae	1	1
*Galago senegalensis*	Galagidae	1	0
*Hylobates lar*	Hylobatidae	5	4
*Chlorocebus sabaeus*	Cercopithecidae	1	0
*Lemur catta*	Lemuridae	20	17
*Leontopithecus rosalia*	Callitrichidae	5	0
*Lophocebus aterrimus*	Cercopithecidae	1	0
*Macaca fuscata*	Cercopithecidae	9	1
*Macaca nigra*	Cercopithecidae	6	6
*Macaca radiata*	Cercopithecidae	1	0
*Mandrillus leucophaeus*	Cercopithecidae	3	2
*Mico argentatus*	Callitrichidae	2	1
*Miopithecus ogouensis*	Cercopithecidae	1	0
*Nomascus gabriellae*	Hylobatidae	7	4
*Otolemur crassicaudatus*	Galagidae	1	1
*Pan troglodytes*	Hominidae	4	2
*Pan troglodytes schweinfurthii*	Hominidae	1	0
*Papio anubis*	Cercopithecidae	1	0
*Pongo pygmaeus*	Hominidae	1	0
*Saguinus imperator*	Callitrichidae	2	2
*Saguinus labiatus*	Callitrichidae	1	0
*Saguinus midas*	Callitrichidae	4	0
*Saimiri sciureus*	Cebidae	18	7
*Symphalangus syndactylus*	Hylobatidae	2	1
*Theropithecus gelada*	Cercopithecidae	1	1
*Varecia rubra*	Lemuridae	11	6

To assess the zoonotic potential of *Giardia*, stool samples were also collected from caregivers directly responsible for the daily care of NHPs included in this study. These individuals are in regular close contact with the NHPs during enclosure cleaning, feeding, and handling. Caregivers used the same sterile sampling kits. The number of caregiver samples per zoo was - 4 from *Na Hrádečku*, 2 from *Hodonín*, 2 from *Děčín*, 4 from *Olomouc*, 5 from *Brno*, and 3 from *Dvůr Králové*. Each participant provided written informed consent and information about the NHP species under their care.

This study was strictly non-invasive and conducted in accordance with animal welfare regulations. All participating zoos approved the research. Human sampling followed the principles of the World Medical Association's Declaration of Helsinki (2013). Personal data were anonymized and processed in accordance with Czech data protection laws. The study protocol was reviewed and approved by the Ethics Committee of the Biology Centre of the Czech Academy of Sciences (Approval No. 2/2020).

All NHP and human samples were transported on ice (4 °C) to the Laboratory of Parasitic Therapy at the Biology Centre of the Czech Academy of Sciences (České Budějovice, Czech Republic) and processed immediately for further analyses.

### 2.2 DNA extraction

Total DNA extraction from fecal samples was performed using the commercial kit CatchGene Stool DNA Kit (CatchGene, New Taipei City, Taiwan) according to manufacturer's instructions. The resulting DNA aliquots, each containing 200 μl, were stored at -20 °C. To prevent contamination, total DNA was processed under sterile conditions in a DNA/RNA UV cleaner box (UVT-B-AR, Biosan, Riga, Latvia). Samples were handled in small sets, with a maximum of 8 samples processed at the same time and from the same zoo, using filtered tips.

### 2.3 Real-time PCR detection of *Giardia intestinalis*

All samples were analyzed using real-time PCR (qPCR) with specific primers and a Taqman probe for *Giardia* detection. We used a modified protocol originally described by Verweij et al. [26] to amplify a 62 bp fragment of the SSU rRNA gene. To increase the reliability of this diagnostic protocol, we confirmed the positivity of samples by sequencing the qPCR amplicons using Sanger method (for more details see Brožová et al. [3]).

All qPCR reactions were performed in 96-well plates using the Light Cycler LC 480 I (Roche, Basel, Switzerland) under following cycling conditions - 95 °C/10 min; 50 × (95 °C / 15 s, 60 °C / 30s, 72 °C / 30s) (for more details see Brožová et al. [3]). Each sample was tested in triplicate to minimize the risk of contamination and to ensure reliability. In cases of ambiguous results, additional replicates were run until a definitive outcome was obtained.

In addition to detection, we quantified the intensity of *Giardia* colonization in all qPCR- positive samples. In our previous study Brožová et al. [3], we optimized a method for absolute quantification of the so-called fecal *Giardia* load using a quantification curve generated from a dilution series of *Giardia* culture (WB ATCC 30957, human isolate; for more details see Brožová et al. [3]). This approach was also applied here to assess the protist load in positive samples.

To assess the co-infections rate of *Giardia* with other protists such as *Blastocystis* sp. and *D. fragilis*, we used data from our previously published study Šejnohová et al. [25]

### 2.4 Genotyping of *Giardia intestinalis*

All qPCR-positive samples were further analyzed using conventional PCR (cPCR) to determine *Giardia* assemblages. We employed an adapted protocol targeting for the triosephosphate isomerase (TPI) gene, originally described by Sulaiman et al. [27] and modified as described in Brožová et al. [3]. The obtained DNA sequences (using Sanger sequencing) were compared with the reference sequences of *G. intestinalis* in the GenBank database by the nucleotide BLASTn program [28]. Sequences were sorted by similarity and aligned using the software Jalview [29].

### 2.5 Testing of DNA inhibition in qPCR-positive samples

To rule out potential inhibition in samples that tested negative by qPCR, we performed an internal control assay. Foreign DNA from experimental rat tissue was added to each sample, and amplification was carried out using a specific qPCR protocol targeting the rat beta-2-microglobulin gene, employing commercially available primers and a TaqMan probe (ThermoFisher Scientific, Waltham, MA, USA). This method followed the approach previously used in Šloufová et al. [30].

### 2.6 Flotation

All fecal samples were also examined coproscopically, using modified Sheather's flotation (specific gravity 1.33) [22].

## 3 Results

### 3.1 Occurrence and genetic diversity of *Giardia intestinalis* in individual zoos

Real-time PCR revealed an overall positivity rate of 45 % (80 out of 179) for *Giardia intestinalis* across all analyzed samples from both NHPs and their caregivers. Specifically, *Giardia* was detected in 47 % (74 out of 159) of NHP samples, representing 24 species (see Table 1), and in 30 % (6 out of 20) of caregiver samples. The distribution of *Giardia* positivity and genotypic diversity across individual zoological gardens is detailed below.

Positivity rates varied considerably between facilities. In *Brno* Zoo, *Giardia* was detected in 37 % of samples (10/27), including one caregiver (1/5) and nine NHPs (9/22). Despite low colonization intensity and the limited sensitivity of cPCR, assemblage B was identified in four NHPs—*Eulemur macaco* (Z152) and *Lemur catta* (Z147, Z149, Z151)—all of which shared identical sequences (Table 2). *Děčín* Zoo exhibited the highest positivity, with 78 % (14/18) of samples testing positive. *Giardia* was found in both caregivers (2/2) and in 12 out of 16 NHPs. However, due to low protist load, assemblage typing was unsuccessful.

**Table 2:** A summary of qPCR results for *Giardia intestinalis* in samples collected from both non-human primates (NHPs) and human caregivers across all Czech zoological gardens.

Zoo place	Sample ID	Host	Ct value	fecal *Giardia* load	Assemblages
*Brno* Zoo	Z152	*Eulemur macaco*	28	10ˇ4	B
	Z147	*Lemur catta*	30⁎	10ˇ4	B
	Z151	*Lemur catta*	30	10ˇ4	B
	Z149	*Lemur catta*	32	10ˇ4	B
	Z150	*Lemur catta*	33	10ˇ2	–
	Z140	*Pan troglodytes*	35	10ˇ2	–
	Z146	*Homo sapiens*	35	10ˇ2	–
	Z148	*Lemur catta*	35	10ˇ2	–
	Z143	*Theropithecus gelada*	36	10ˇ2	–
	Z145	*Saimiri sciureus*	36	10ˇ2	–
*Děčín* Zoo	Z36	*Varecia rubra*	31	10ˇ3	–
	Z35	*Varecia rubra*	33	10ˇ3	–
	Z38	*Ateles geoffroyi vellerosus*	33	10ˇ3	–
	Z40	*Ateles geoffroyi vellerosus*	33	10ˇ3	–
	Z25	*Macaca nigra*	34	10ˇ3	–
	Z28	*Homo sapiens*	34	10ˇ3	–
	Z34	*Varecia rubra*	34⁎	10ˇ3	–
	Z27	*Homo sapiens*	36	10ˇ2	–
	Z29	*Macaca nigra*	36⁎	10ˇ2	–
	Z30	*Macaca nigra*	36⁎	10ˇ2	–
	Z32	*Macaca nigra*	36⁎	10ˇ2	–
	Z33	*Macaca nigra*	36⁎	10ˇ2	–
	Z37	*Varecia rubra*	36	10ˇ2	–
	Z31	*Macaca nigra*	38⁎	10ˇ1	–
*Dvůr Králové* Zoo	Z173	*Mandrillus leucophaeus*	24	10ˇ5	B
	Z168	*Otolemur crassicaudatus*	35⁎	10ˇ2	–
	Z169	*Colobus angolensis*	36	10ˇ1	–
	Z167	*Cercopithecus nictitans*	37	10ˇ1	–
	Z172	*Mandrillus leucophaeus*	37	10ˇ1	–
*Hodonín* Zoo	Z22	*Varecia rubra*	32	10ˇ4	A1
	Z19	*Gibon lar*	35⁎	10ˇ2	–
	Z20	*Eulemur macaco*	35	10ˇ2	–
	Z21	*Pan troglodytes*	35	10ˇ2	–
	Z17	*Homo sapiens*	37	10ˇ1	–
*Na Hrádečku* Zoo	Z9	*Lemur catta*	21	10ˇ7	B
	Z14	*Callithrix jacchus*	31	10ˇ4	B
	Z10	*Callithrix penicillata*	32	10ˇ3	–
	Z15	*Mico argentatus*	33	10ˇ3	–
	Z11	*Callithrix penicillata*	34	10ˇ3	–
	Z2	*Homo sapiens*	36	10ˇ2	–
	Z4	*Homo sapiens*	36	10ˇ2	–
	Z6	*Eulemur rufifrons*	36	10ˇ2	–
*Olomouc* Zoo	Z41	*Lemur catta*	32	10ˇ4	B
	Z82	*Saimiri sciureus*	32	10ˇ4	A1
	Z88	*Lemur catta*	32	10ˇ4	B
	Z47	*Macaca fuscata*	33	10ˇ3	–
	Z54	*Lemur catta*	33	10ˇ3	–
	Z68	*Lemur catta*	33	10ˇ2	–
	Z70	*Eulemur macaco*	33	10ˇ2	–
	Z73	*Callithrix pygmaea*	33⁎	10ˇ2	–
	Z136	*Lemur catta*	33	10ˇ2	–
	Z69	*Nomascus gabriellae*	34	10ˇ2	–
	Z77	*Saimiri sciureus*	34	10ˇ2	–
	Z85	*Callithrix penicillata*	34	10ˇ2	–
	Z123	*Lemur catta*	34	10ˇ2	–
	Z42	*Symphalangus syndactylus*	35	10ˇ2	–
	Z60	*Hylobates lar*	35	10ˇ2	–
	Z66	*Hylobates lar*	35	10ˇ2	–
	Z100	*Erythrocebus patas*	35	10ˇ2	–
	Z104	*Saimiri sciureus*	35	10ˇ2	–
	Z106	*Callithrix penicillata*	35	10ˇ2	–
	Z109	*Callimico goeldii*	35	10ˇ2	–
	Z137	*Callithrix penicillata*	35	10ˇ2	–
	Z44	*Eulemur macaco*	36	10ˇ2	–
	Z45	*Nomascus gabriellae*	36	10ˇ2	–
	Z50	*Varecia rubra, Eulemur macaco*	36	10ˇ2	–
	Z55	*Erythrocebus patas*	36	10ˇ2	–
	Z97	*Lemur catta*	36	10ˇ1	–
	Z98	*Lemur catta*	36	10ˇ2	–
	Z115	*Nomascus gabriellae*	36	10ˇ2	–
	Z121	*Saimiri sciureus*	36	10ˇ2	–
	Z124	*Saimiri sciureus*	36	10ˇ2	–
	Z126	*Saguinus imperator*	36	10ˇ2	–
	Z129	*Saguinus imperator*	36	10ˇ2	–
	Z58	*Nomascus gabriellae*	37	10ˇ2	–
	Z99	*Lemur catta*	37	10ˇ1	–
	Z101	*Lemur catta*	37	10ˇ2	–
	Z107	*Saimiri sciureus*	37	10ˇ1	–
	Z118	*Lemur catta*	37	10ˇ1	–
	Z128	*Hylobates lar*	37	10ˇ1	–

⁎ Sample in which *Giardia* cysts were detected using Sheathers' flotation.

In *Dvůr Králové* Zoo, the positivity rate was 33 % (5/15), with five positive NHPs (5/12) and no detection in caregivers (0/3). Assemblage B was identified in a single case from *Mandrillus leucophaeus* (Z173). *Hodonín* Zoo showed a high positivity of 71 % (5/7), including one caregiver (1/2) and four NHPs (4/5). Assemblage A1 was detected in *Varecia rubra* (Z22). In *Na Hrádečku* Zoo, 53 % (8/15) of samples tested positive, including two caregivers (2/4) and six NHPs (6/11). Assemblage B was found in L. *catta* and in *Callithrix jacchus* (Z9, Z14). In *Olomouc* Zoo, the positivity was 39 % (38/97), with *Giardia* detected in 38 NHPs (38/93) and none of the caregivers were positive (0/4) (Table 2). Assemblage A1 was identified in *Saimiri sciureus* (Z82) and assemblage B in L. *catta* (Z41 and Z88).

No evidence of internal inhibition was detected in any of the samples using foreign DNA. Additionally, all samples underwent testing using Sheather's flotation method, and only ten samples tested positive (more details in Table 2).

### 3.2 *Giardia* fecal load in NHPs and their caregivers

The intensity of *Giardia* colonization in human and NHP samples varied significantly (Table 3). Most samples (57 out of 179) had an estimated fecal *Giardia* load between 10^1^ and 10^2^ with a Ct value between 34 and 38. A smaller proportion of samples (21 out of 179) had a fecal *Giardia* load in the range of 10^3^ to 10^4^, with a Ct value between 28 and 33. Heavy colonization was only found in two samples only, with an estimated fecal *Giardia* load of 10^5^ to 10^7^, characterized by Ct values between 21 and 24. All colonizations in the caregivers were low with a fecal *Giardia* load in the range of 10^1^–10^3^, with a Ct value between 34 and 37. For more details about correlation fecal *Giardia* load and Ct values see Brožová et al. [3].

**Table 3:** Evaluation of fecal *Giardia* load in NHPs and caregiver samples based on the established quantification curve (set in the range of 10^−1^ to 10^5^ cells per 1 qPCR reaction).

Fecal *Giardia* load	Number of positive samples/ Number of all samples	Ct value range
10^1^–10^2^	57/179	34–38
10^3^–10^4^	21/179	28–33
10^5^–10^7^	2/179	21–24

### 3.3 Assemblage identification

With the currently used protocols, we cannot detect *Giardia* assemblages in qPCR-positive samples with a colonization intensity below the threshold of 10^4^ cells per reaction (for more details, see Brožová et al. [3]). As a result, we obtained assemblage data from only 11 positive samples of NHPs, all of which had a fecal load of over, all with fecal loads above 10^4^ (Table 2). Nine of these belonged to assemblage B (Z9, Z14, Z41, Z88, Z147, Z149, Z151, Z152, Z173) found in L. *catta*, *E. macaco*, *C. jacchus* and *M. leucophaeus*, and two cases of assemblage A1 (Z22 and Z82) in *V. rubra* and *S. sciureus* (Table 2). We did not obtain information about assemblages from any of the caregivers, as all six positive caregivers had fecal loads ranging from 10^1^ to 10^3^.

Sequences obtained in this study were deposited in GenBank under accession numbers PV544239 - PV544249.

### 3.4 Zoonotic transmission between NHPs and their caregivers

In one zoo, we found identical sequences of assemblages B in *E. macaco* (Z152) and three cases of L. *catta* (Z147, Z149 and Z151). The male *E. macaco* shared indoor and outdoor enclosures with L. *catta*. All NHPs in this enclosure were tested positive for *Giardia* using qPCR. However, other L. *catta* exhibited low colonization levels (fecal loads of 10^2^–10^3^), below the sequencing threshold of 10^4^. These NHPs were all cared for by the same caregiver (Z146), who also tested positive for *Giardia*. However, the caregiver's low colonization level (fecal load of 10^2^) limited the sequencing and assessment of potential zoonotic transmission (Fig. 2).

### 3.5 Co-infection of *Giardia intestinalis* with other protists

**Fig. 1:**
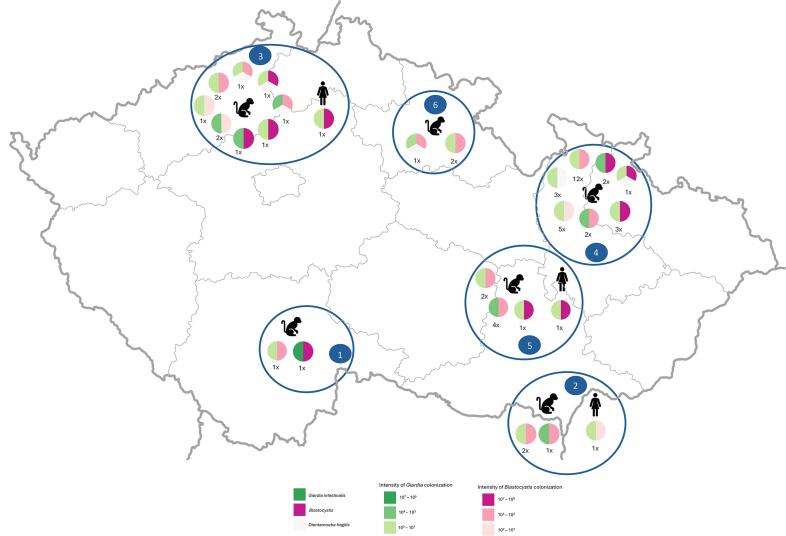
Co-infections of *Giardia intestinalis* with *Blastocystis* sp. and *Dientamoeba fragilis* across all six Czech zoological gardens. x means same level of intensity of colonization multiple times. The coordinates for the individual zoos are as follows: (1) *Na Hrádečku* Zoo (49.1083500 N, 15.0314128E), (2) *Hodonín* Zoo (48.8642753 N, 17.1077775E), (3) *Děčín* Zoo (50.7797175 N, 14.1973347E), (4) *Olomouc* Zoo (49.6345464 N, 17.3408561E), (5) *Brno* Zoo (49.2307794 N, 16.5335656E), and (6) *Dvůr Králové* Zoo (50.4340289 N, 15.7989708E).

In addition to *G. intestinalis*, co-infections with other intestinal protists previously described in Šejnohová et al. [25] were frequently observed in both NHPs and caregivers (Fig. 1; Supplementary Data 1). The most common co-infection involved *Blastocystis* sp. (Supplementary Data 2), detected in 53 cases, including three caregivers. In the majority of these cases (43/53), the fecal load of *Blastocystis* exceeded that of *Giardia*, while in four cases *Giardia* was dominant and in six cases the loads were comparable. Co-infection of *Giardia* with *D. fragilis* was identified in eight NHPs. Additionally, five NHPs harbored all three protists (Supplementary Data 3). Quantification of colonization intensity for *D. fragilis*-positive samples was not possible, as no culture material was available for standardization [25].

**Fig. 2:**
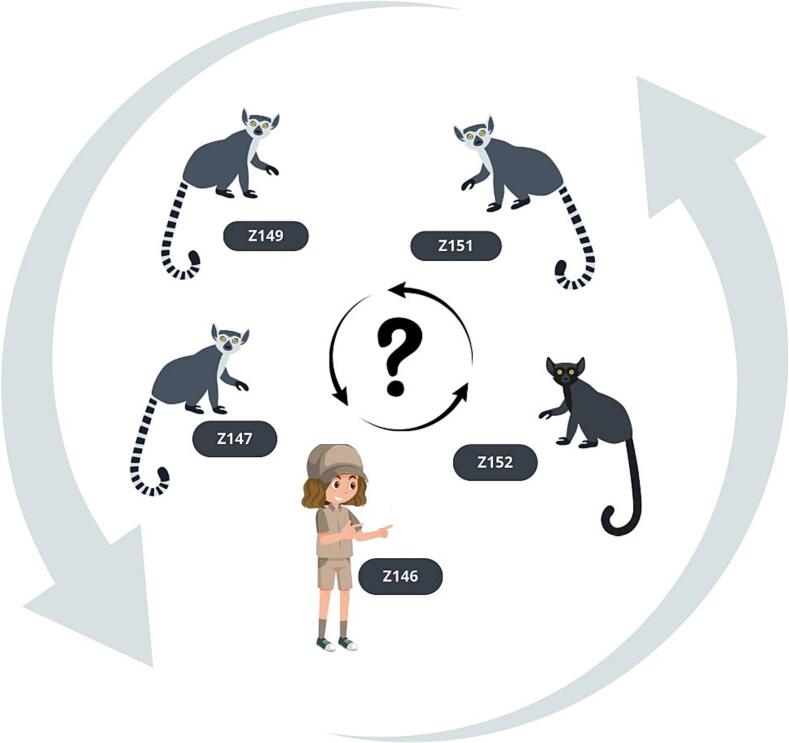
Possible transmission of *Giardia intestinalis* between a caregiver and the non-human primates (NHPs) under caregiver's care. *Eulemur macaco* (Z152) and *Lemur catta* (Z147, Z149, Z151) shared identical sequences of *Giardia* assemblage B, suggesting a common source of infection, likely due to their shared indoor and outdoor spaces. Although their caregiver (Z146) was also tested *Giardia* positive by qPCR, the fecal load was below the threshold of 10^4^, so obtaining sequences and assessing zoonotic transmission was not possible.

## 4 Discussion

The zoonotic transmission of intestinal protists between humans and non-human primates (NHPs) is a key aspect of the One Health concept and has attracted growing attention [31]. Zoological gardens provide a unique environment for studying these interactions, as the daily close contact between NHPs and their caregivers creates favorable conditions for cross-species transmission [14,19,20]. These facilities may serve as hotspots for human–NHP transmission of *Giardia intestinalis*, an intestinal opportunistic protist widely reported in captive NHPs worldwide (e.g., [12,14,18,19,33]). Motivated by this context, we conducted a molecular survey in six Czech zoological gardens to determine the prevalence of *G. intestinalis* in both NHPs and their caregivers. Our recent findings further indicate that colonization in gut-healthy humans is often asymptomatic [3], suggesting that silent carriers may contribute to unnoticed transmission and pose a risk to immunocompromised individuals.

Across six Czech zoos, qPCR analysis revealed a *Giardia* prevalence of 47 % in NHPs and 30 % in their caregivers. These findings confirm that this intestinal protist is common among captive NHPs and also present at a notable level in humans who work in close daily contact with them. Comparable prevalence has been reported in other European studies. Köster et al. [15] found 22 % of NHPs and 11 % of caregivers *Giardia*-positive at Córdoba Zoo in Spain using the same qPCR protocol, while a broader survey across zoos in Spain, Germany and France detected 16 % in NHPs and 1.4 % in caregivers [19]. Overall, prevalence reported in NHPs ranges from 17 % in Slovakia to 33 % in Italy and up to 70 % in two Spanish zoos [9,16,18]. Taken together, these data strengthen the evidence that close human–NHP contact in zoological settings can facilitate *Giardia* transmission, most probably in both directions.

Building on these overall findings, our study also revealed substantial variation in prevalence among individual zoos, ranging from 33 % in *Dvůr Králové* Zoo to 78 % in *Děčín* Zoo. Among NHPs, prevalence ranged from 41 % to 80 %, while in caregivers it ranged from 0 % to 100 %. Such differences likely reflect local conditions, including water sources, environmental contamination, animal density, and the frequency of close contact between NHPs and caregivers. Interestingly, the highest prevalence in both humans and NHPs was observed in *Děčín* and *Hodonín* zoos, which house the fewest NHPs. This pattern suggests that close and repeated contact facilitates *Giardia* transmission and may lead to the development of partial immunity, resulting in asymptomatic carriage [32]. A similar trend—characterized by low infection intensity and frequent contact with *Giardia*-positive animals—was also observed in our previous study Brožová et al. [3]. Such individuals can still harbor the protist and, despite being symptom-free, may only test positive with highly sensitive molecular methods such as qPCR. This finding is consistent with mechanistic evidence indicating that partial immunity against *Giardia* is inherently limited by the protist's immune evasion strategies, such as antigenic variation and the induction of transient antibody responses [32]. These observations warrant further investigation into the role of asymptomatic carriers in *Giardia* transmission.

Among the examined NHP species, *Giardia* was detected in 24 of 37 species across all zoos, confirming its widespread presence in diverse primate hosts. The highest prevalence occurred in *Macaca nigra* (100 %), *Lemur catta* (85 %), and *Hylobates lar* (80 %). As these species showed the highest infection rates, we examined their prevalence across individual zoos in more detail. In L. *catta*, prevalence ranged from 0 % in *Dvůr Králové* to 100 % in *Brno* and *Na Hrádečku*, consistent with previous reports of 38–89 % [12,18,19,34]. Notably, Fomsgaard et al. [12] reported a significantly higher prevalence in zoo-housed L. *catta* (88.6 %) compared to wild populations in Madagascar (20 %). In *M. nigra* and *H. lar*, prevalence reached 100 % in *Děčín* Zoo and 75–100 % in *Olomouc* and *Hodonín* Zoos. Other studies have generally reported lower rates (e.g., [[35], [36], [37]]), likely reflecting methodological differences. For example, Tangtrongsup et al. [36] and Wulandari et al. [37] used flotation techniques, which can underestimate prevalence due to intermittent cyst shedding. A similar pattern appeared in our study when flotation detected *Giardia* cysts in only 10 cases, whereas qPCR identified 80 positive samples.

To understand the epidemiology of *G. intestinalis*, absolute quantification of colonization intensity by qPCR—referred to as fecal protist load [30]—is essential. In the present study, most positive samples exhibited very low fecal loads (10^1^−10^2^), corresponding to Ct values of 34–38. As we demonstrated previously in Brožová et al. [3], qPCR can reliably detect *Giardia* loads as low as 10^1^, whereas conventional PCR targeting the triosephosphate isomerase (TPI) gene, including nested PCR, consistently requires fecal loads above 10^4^ to yield an amplicon. Consequently, assemblage determination was not feasible for all qPCR-positive samples, underscoring the need for more sensitive genotyping methods.

Among the available genotyping target genes, we selected the TPI gene, which showed the highest specificity and sensitivity in our previous study (Brožová et al. [3]). Other genes consistently underperformed and often failed to amplify *Giardia* DNA even in microscopically positive samples. In the current dataset, genotyping succeeded in only 14 % of qPCR-positive NHP samples, all with high fecal loads (10^4^–10^7^). These samples revealed assemblage A in *Saimiri sciureus* and *Varecia rubra*, and assemblage B in L. *catta*, *Eulemur macaco*, *Mandrillus leucophaeus*, and *Callithrix jacchus*.

Fecal *Giardia* loads in human caregivers were similarly low (10^1^−10^3^), and no sequences could be obtained to confirm zoonotic transmission. A comparable limitation was reported by Köster et al. [19], who successfully genotyped only 22 % of positive samples, all with Ct values below 30, which aligns with our findings.

Due to the inability to genotype samples with low colonization levels, zoonotic transmission could not be fully assessed, leaving potential transmission pathways hypothetical. An illustrative case occurred in one zoo, where *Giardia* was detected in a caregiver and several NHPs under his care. Identical assemblage B was identified in *E. macaco* and three L. *catta*, all housed together, suggesting a common source of infection. All NHPs in this enclosure were qPCR-positive, but both the caregiver and the other L. *catta* exhibited low colonization levels (10^2^−10^3^). Further investigation revealed that the caregiver's dog had previously been diagnosed with *Giardia*. To explore this link, fecal samples from both the caregiver and the dog were collected over three consecutive days. qPCR detected only very low *Giardia* loads, likely remnants of a past infection. These findings suggest possible bidirectional transmission between lemurs and the caregiver, but low infection levels hindered genotyping. This limitation underscores the need to clarify *Giardia* transmission dynamics, for which whole-genome sequencing could provide higher resolution and deeper epidemiological insight [3,20].

Since we had already published data on the prevalence of *Blastocystis* sp. and *Dientamoeba fragilis* in the same samples [25], we were able to assess co-infections involving all three intestinal protists. Co-infection of *Giardia* with *Blastocystis* sp. was the most frequent (66 %), while co-infection with *Dientamoeba* occurred in 10 % of cases. Triple infection with all three protists was detected in 6 % of samples. Previous studies reported lower *Giardia*–*Blastocystis* co-infection rates in NHPs (8–13 %) [15,19], and to our knowledge, *Giardia–Dientamoeba* co-infection has not yet been documented in NHPs.

## 5 Conclusion

Our study provides new insight into the epidemiology of *G. intestinalis* in zoological settings, revealing high prevalence in both NHPs and their caregivers and highlighting the potential for human–NHP transmission. The findings underscore the role of species- and location-specific factors, as well as the importance of monitoring asymptomatic colonization in One Health contexts.

Limited genotyping success, mainly due to low fecal loads, illustrates the need for more sensitive molecular approaches to clarify transmission pathways and assess zoonotic risk. Strengthening surveillance and improving diagnostic strategies will be essential for mitigating *Giardia* transmission and informing evidence-based management in zoos.

## CRediT authorship contribution statement

**Kristýna Brožová:** Writing – review & editing, Writing – original draft, Visualization, Validation, Software, Project administration, Methodology, Investigation, Formal analysis, Data curation. **Anna Šejnohová:** Investigation. **Monika Koutenská:** Investigation. **Zuzana Pavlíčková:** Methodology. **Milan Jirků:** Methodology. **Eliška Zimmelová:** Investigation. **Oldřiška Kadlecová:** Investigation. **Klára J. Petrželková:** Project administration, Conceptualization. **Kateřina Jirků:** Writing – review & editing, Validation, Project administration, Funding acquisition, Conceptualization.

## Ethics statement

The studies involving human participants were reviewed and approved by the Ethics Committee of the Biology Center of the Czech Academy of Sciences (reference number: 2/2020). Written informed consent to participate in this study was provided by the participants' legal guardian/next of kin. All data were anonymized and processed according to the applicable laws of the Czech Republic (e.g., Act no. 101/2000 Coll and subsequent regulations). In case of the rat tissue used for testing of the internal inhibition, we used samples from the experiment approved by the Committee on the Ethics of Animal Experiments of the Biology Centre of the Czech Academy of Sciences (České Budějovice, permit no. 33/2018) and by the Resort Committee of the Czech Academy of Sciences (Prague, Czech Republic) according to strict accordance with Czech legislation (Act No. 166/1999 Coll. on veterinary care and on changes of some related laws, and Act No. 246/1992 Coll. on the protection of animals against cruelty), as well as the legislation of the European Union.

## Funding

This work was financially supported by a grant from the 10.13039/501100001824Czech Science Foundation (22-04837S) to KJ.

## Declaration of competing interest

Authors declare no conflict of interest.

## Data Availability

The data are available in the GenBank database under these accession numbers [PV544239 - PV544249].

## Appendix A Supplementary data

Supplementary material Image 1
