# Supplementary material for: Epidemiology of Giardia intestinalis in non-human primates and their caregivers: A study from Czech zoos
Source: One Health. 2025 Aug 19;21:101176. doi: 10.1016/j.onehlt.2025.101176 (PMC12765084; doi:10.1016/j.onehlt.2025.101176)
Supplement: Supplementary file 1 — Supplementary material [file mmc1.docx]

**Supplementary data:**

**SD1.** Co-infections of *Giardia intestinalis* [GI] with other protists, specifically *Blastocystis* [Bl] and *Dientamoeba fragilis* [DF].

| **Sample** | **GI** | **Bl** | **DF** | **Sample** | **GI** | **Bl** | **DF** | **Sample** | **GI** | **Bl** | **DF** | **Sample** | **GI** | **Bl** | **DF** |
| --- | --- | --- | --- | --- | --- | --- | --- | --- | --- | --- | --- | --- | --- | --- | --- |
| Z2 | + | - | - | Z33 | + | + | - | Z70 | + | - | - | Z126 | + | - | - |
| Z4 | + | - | - | Z34 | + | + | + | Z73 | + | - | - | Z128 | + | + | - |
| Z6 | + | + | - | Z35 | + | + | - | Z77 | + | - | - | Z129 | + | - | - |
| Z9 | + | + | - | Z36 | + | + | - | Z82 | + | - | - | Z136 | + | + | - |
| Z10 | + | - | - | Z37 | + | + | - | Z85 | + | - | - | Z137 | + | - | + |
| Z11 | + | - | - | Z38 | + | - | - | Z88 | + | + | - | Z140 | + | - | - |
| Z14 | + | - | - | Z40 | + | - | - | Z97 | + | + | - | Z143 | + | + | - |
| Z15 | + | - | - | Z41 | + | + | - | Z98 | + | + | - | Z145 | + | - | - |
| Z17 | + | + | - | Z42 | + | + | - | Z99 | + | + | - | Z146 | + | + | - |
| Z19 | + | + | - | Z44 | + | + | + | Z100 | + | + | - | Z147 | + | + | - |
| Z20 | + | - | - | Z45 | + | + | - | Z101 | + | + | - | Z148 | + | + | - |
| Z21 | + | + | - | Z47 | + | + | - | Z104 | + | - | + | Z149 | + | + | - |
| Z22 | + | + | - | Z50 | + | + | - | Z106 | + | - | - | Z150 | + | + | - |
| Z25 | + | + | - | Z54 | + | + | - | Z107 | + | - | - | Z151 | + | + | - |
| Z27 | + | + | - | Z55 | + | + | - | Z109 | + | + | - | Z152 | + | + | - |
| Z28 | + | - | - | Z58 | + | + | - | Z115 | + | + | - | Z167 | + | + | + |
| Z29 | + | + | - | Z60 | + | + | - | Z118 | + | + | - | Z168 | + | - | - |
| Z30 | + | + | + | Z66 | + | + | - | Z121 | + | - | - | Z169 | + | + | - |
| Z31 | + | + | + | Z68 | + | + | - | Z123 | + | + | - | Z172 | + | + | - |
| Z32 | + | + | - | Z69 | + | + | - | Z124 | + | - | + | Z173 | + | - | - |

**SD2: Co-infection of *Giardia intestinalis* and *Blastocystis* sp.**

| **Zoo place** | **Sample ID** | **Host** | ***Giardia intestinalis*** | | |  | ***Blastocystis* sp.** | | |
| --- | --- | --- | --- | --- | --- | --- | --- | --- | --- |
|  |  |  | **Ct value** | **Fecal load** | **Assemblages** |  | **Ct**  **value** | **Fecal load** | **Subtypes** |
| ***Na Hrádečku* Zoo** | Z6 | *Eulemur rufifrons* | 36 | 10ˇ2 | - |  | 28 | 10ˇ4 | ST1 (100%) |
|  | Z9 | *Lemur catta* | 21 | 10ˇ7 | B |  | 21 | 10ˇ6 | ST5 (100%) |
| ***Hodonín* Zoo** | Z17 | *Homo sapiens* | 37 | 10ˇ1 | - |  | 32 | 10ˇ1 | - |
|  | Z19 | *Gibon lar* | 35 | 10ˇ2 | - |  | 25 | 10ˇ4 | ST1 (53%), ST2 (47%) |
|  | Z21 | *Pan troglodytes* | 35 | 10ˇ2 | - |  | 24 | 10ˇ4 | ST47 (88%), ST48 (4%) |
|  | Z22 | *Varecia rubra* | 32 | 10ˇ4 | A1 |  | 22 | 10ˇ4 | ST4 (100%) |
| ***Děčín* Zoo** | Z25 | *Macaca nigra* | 34 | 10ˇ3 | - |  | 30 | 10ˇ2 | ST1 (18%), ST3 (82%) |
|  | Z27 | *Homo sapiens* | 36 | 10ˇ2 | - |  | 21 | 10ˇ5 | ST1 (89%) ST3 (11%) |
|  | Z29 | *Macaca nigra* | 36 | 10ˇ2 | - |  | 24 | 10ˇ4 | ST1 (76%), ST3 (24%) |
|  | Z30 | *Macaca nigra* | 36 | 10ˇ2 | - |  | 24 | 10ˇ4 | ST1 (73%), ST3 (27%) |
|  | Z31 | *Macaca nigra* | 38 | 10ˇ1 | - |  | 22 | 10ˇ5 | ST1 (70%), ST3 (30%) |
|  | Z32 | *Macaca nigra* | 36 | 10ˇ2 | - |  | 25 | 10ˇ4 | ST1 (62%), ST3 (38%) |
|  | Z33 | *Macaca nigra* | 36 | 10ˇ2 | - |  | 22 | 10ˇ5 | ST1 (50%), ST3 (50%) |
|  | Z34 | *Varecia rubra* | 34 | 10ˇ3 | - |  | 26 | 10ˇ3 | ST1 (54%), ST5 (1%), ST8 (45%) |
|  | Z35 | *Varecia rubra* | 33 | 10ˇ3 | - |  | 22 | 10ˇ5 | ST1 (90%), ST8 (10%) |
|  | Z36 | *Varecia rubra* | 31 | 10ˇ3 | - |  | 32 | 10ˇ2 | - |
|  | Z37 | *Varecia rubra* | 36 | 10ˇ2 | - |  | 32 | 10ˇ2 | ST1 (100%) |
| ***Olomouc* Zoo** | Z41 | *Lemur catta* | 32 | 10ˇ4 | B |  | 25 | 10ˇ4 | ST2 (90%), ST8 (9%) |
|  | Z42 | *Symphalangus syndactylus* | 35 | 10ˇ2 | - |  | 24 | 10ˇ4 | ST2 (97%), ST3 (3%) |
|  | Z44 | *Eulemur macaco* | 36 | 10ˇ2 | - |  | 23 | 10ˇ5 | ST1 (100%) |
|  | Z45 | *Nomascus gabriellae* | 36 | 10ˇ2 | - |  | 30 | 10ˇ3 | ST1 (94%), ST8 (5%) |
|  | Z47 | *Macaca fuscata* | 33 | 10ˇ3 | - |  | 20 | 10ˇ6 | ST1 (17%), ST2 (52%), ST3 (31%) |
|  | Z50 | *Varecia + Eulemur* | 36 | 10ˇ2 | - |  | 27 | 10ˇ3 | ST2 (100%) |
|  | Z54 | *Lemur catta* | 33 | 10ˇ3 | - |  | 22 | 10ˇ6 | ST2 (68%), ST8 (32%) |
|  | Z55 | *Erythrocebus patas* | 36 | 10ˇ2 | - |  | 25 | 10ˇ5 | ST3 (100%) |
|  | Z58 | *Nomascus gabriellae* | 37 | 10ˇ2 | - |  | 22 | 10ˇ5 | ST8 (21%) |
|  | Z60 | *Hylobates lar* | 35 | 10ˇ2 | - |  | 28 | 10ˇ3 | ST1 (100%) |
|  | Z66 | *Hylobates lar* | 35 | 10ˇ2 | - |  | 26 | 10ˇ4 | ST1 (100%) |
|  | Z68 | *Lemur catta* | 33 | 10ˇ2 | - |  | 24 | 10ˇ4 | ST2 (58%), ST8 (42%) |
|  | Z69 | *Nomascus gabriellae* | 34 | 10ˇ2 | - |  | 31 | 10ˇ2 | ST1 (90%), ST8 (10%) |
|  | Z88 | *Lemur catta* | 32 | 10ˇ4 | B |  | 26 | 10ˇ3 | ST2 (100%) |
|  | Z97 | *Lemur catta* | 36 | 10ˇ1 | - |  | 29 | 10ˇ2 | - |
|  | Z98 | *Lemur catta* | 36 | 10ˇ2 | - |  | 27 | 10ˇ3 | ST2 (65%), ST8 (35%) |
|  | Z99 | *Lemur catta* | 37 | 10ˇ1 | - |  | 29 | 10ˇ2 | ST2 (99%), ST8 (1%) |
|  | Z100 | *Erythrocebus patas* | 35 | 10ˇ2 | - |  | 24 | 10ˇ4 | ST1 (81%), ST3 (19%) |
|  | Z101 | *Lemur catta* | 37 | 10ˇ2 | - |  | 24 | 10ˇ4 | ST2 (56%), ST8 (44%) |
|  | Z109 | *Callimico goeldii* | 35 | 10ˇ2 | - |  | 32 | 10ˇ2 | ST2 (100%) |
|  | Z115 | *Nomascus gabriellae* | 36 | 10ˇ2 | - |  | 27 | 10ˇ3 | ST1 (97%), ST8 (3%) |
|  | Z118 | *Lemur catta* | 37 | 10ˇ1 | - |  | 25 | 10ˇ4 | ST2 (53%), ST8 (47%) |
|  | Z123 | *Lemur catta* | 34 | 10ˇ2 | - |  | 26 | 10ˇ3 | ST2 (37%), ST8 (63%) |
|  | Z128 | *Hylobates lar* | 37 | 10ˇ1 | - |  | 32 | 10ˇ2 | ST3 (100%) |
|  | Z136 | *Lemur catta* | 33 | 10ˇ2 | - |  | 25 | 10ˇ4 | ST2 (70%), ST8 (30%) |
| ***Brno***  **Zoo** | Z143 | *Theropithecus gelada* | 36 | 10ˇ2 | - |  | 24 | 10ˇ4 | ST1 (65%), ST2 (35%) |
|  | Z146 | *Homo sapiens* | 35 | 10ˇ2 | - |  | 21 | 10ˇ5 | ST7 (100%) |
|  | Z147 | *Lemur catta* | 30 | 10ˇ4 | B |  | 24 | 10ˇ4 | ST5 (100%) |
|  | Z148 | *Lemur catta* | 35 | 10ˇ2 | - |  | 24 | 10ˇ4 | ST5 (100%) |
|  | Z149 | *Lemur catta* | 32 | 10ˇ4 | B |  | 24 | 10ˇ4 | ST5 (100%) |
|  | Z150 | *Lemur catta* | 33 | 10ˇ2 | - |  | 20 | 10ˇ5 | ST5 (100%) |
|  | Z151 | *Lemur catta* | 30 | 10ˇ4 | B |  | 24 | 10ˇ4 | ST5 (100%) |
|  | Z152 | *Eulemur macaco* | 28 | 10ˇ4 | B |  | 19 | 10ˇ5 | ST5 (100%) |
|  | Z167 | *Cercopithecus nictitans* | 37 | 10ˇ1 | - |  | 24 | 10ˇ4 | ST1 (65%), ST3 (34%) |
|  | Z169 | *Colobus angolensis* | 36 | 10ˇ1 | - |  | 27 | 10ˇ2 | ST8 (100%) |
|  | Z172 | *Mandrillus leucophaeus* | 37 | 10ˇ1 | - |  | 23 | 10ˇ4 | ST1 (47%), ST3 (52%) |

**SD3:** An overview of the occurrence three intestinal protist *Giardia intestinalis*, *Blastocystis* sp. and *Dientamoeba fragilis* among NHPs family.

| **Host species** | **Family** | **Number of samples** | **Prevalence of *G. intestinalis*** | **Prevalence of *Blastocystis* sp.** | **Prevalence of *D. fragilis*** |  |
| --- | --- | --- | --- | --- | --- | --- |
|  |  |  |  |  |  |  |
| *Ateles geoffroyi vellerosus* | Atelidae | 4 | 50% (2/4) | 0% (0/4) | 0% (0/4) |  |
| *Callimico goeldii* | Callitrichidae | 2 | 50% (1/2) | 100% (2/2) | 0% (0/2) |  |
| *Callithrix jacchus* | Callitrichidae | 1 | 100% (1/1) | 0% (0/1) | 0% (0/1) |  |
| *Callithrix penicillata* | Callitrichidae | 13 | 38% (5/13) | 0% (0/13) | 7,7% (1/13) |  |
| *Callithrix pygmaea* | Callitrichidae | 2 | 50% (1/2) | 0% (0/2) | 0% (0/2) |  |
| *Cercopithecus campbelli* | Cercopithecidae | 1 | 0% (0/1) | 0% (0/1) | 0% (0/1) |  |
| *Cercopithecus nictitans* | Cercopithecidae | 1 | 100% (1/1) | 100% (1/1) | 100% (1/1) |  |
| *Colobus angolensis* | Cercopithecidae | 2 | 50% (1/2) | 100% (2/2) | 0% (0/2) |  |
| *Erythrocebus patas* | Cercopithecidae | 6 | 33% (2/6) | 66.7% (4/6) | 0% (0/6) |  |
| *Eulemur albifrons* | Lemuridae | 6 | 0% (0/6) | 66.7% (4/6) | 0% (0/6) |  |
| *Eulemur macaco* | Lemuridae | 11 | 36% (4/11) | 54.5% (6/11) | 9.1% (1/11) |  |
| *Eulemur rufifrons* | Lemuridae | 1 | 100% (1/1) | 100% (1/1) | 0% (0/1) |  |
| *Galago senegalensis* | Galagidae | 1 | 0% (0/1) | 0% (0/1) | 0% (0/1) |  |
| *Hylobates lar* | Hylobatidae | 5 | 80% (4/5) | 100% (5/5) | 20% (1/5) |  |
| *Chlorocebus sabaeus* | Cercopithecidae | 1 | 0% (0/1) | 100% (1/1) | 0% (0/1) |  |
| *Lemur catta* | Lemuridae | 20 | 85% (17/20) | 95% (19/20) | 0% (0/20) |  |
| *Leontopithecus rosalia* | Callitrichidae | 5 | 0% (0/5) | 0% (0/5) | 20% (1/5) |  |
| *Lophocebus aterrimus* | Cercopithecidae | 1 | 0% (0/1) | 100% (1/1) | 0% (0/1) |  |
| *Macaca fuscata* | Cercopithecidae | 9 | 11% (1/9) | 100% (9/9) | 0% (0/9) |  |
| *Macaca nigra* | Cercopithecidae | 6 | 100% (6/6) | 100% (6/6) | 33.3% (2/6) |  |
| *Macaca radiata* | Cercopithecidae | 1 | 0% (0/1) | 100% (1/1) | 0% (0/1) |  |
| *Mandrillus leucophaeus* | Cercopithecidae | 3 | 67% (2/3) | 66.7% (2/3) | 0% (0/3) |  |
| *Mico argentatus* | Callitrichidae | 2 | 50% (1/2) | 0% (0/2) | 0% (0/2) |  |
| *Miopithecus ogouensis* | Cercopithecidae | 1 | 0% (0/1) | 100% (1/1) | 0% (0/1) |  |
| *Nomascus gabriellae* | Hylobatidae | 7 | 57% (4/7) | 85.7% (6/7) | 0% (0/7) |  |
| *Otolemur crassicaudatus* | Galagidae | 1 | 100% (1/1) | 0% (0/1) | 0% (0/1) |  |
| *Pan troglodytes* | Hominidae | 4 | 50 % (2/4) | 75% (3/4) | 0% (0/4) |  |
| *Pan troglodytes schweinfurthii* | Hominidae | 1 | 0% (0/1) | 100% (1/1) | 0% (0/1) |  |
| *Papio anubis* | Cercopithecidae | 1 | 0% (0/1) | 100% (1/1) | 0% (0/1) |  |
| *Pongo pygmaeus* | Hominidae | 1 | 0% (0/1) | 100% (1/1) | 0% (0/1) |  |
| *Saguinus imperator* | Callitrichidae | 2 | 100% (2/2) | 0% (0/2) | 0% (0/2) |  |
| *Saguinus labiatus* | Callitrichidae | 1 | 0% (0/1) | 0% (0/1) | 0% (0/1) |  |
| *Saguinus midas* | Callitrichidae | 4 | 0% (0/4) | 0% (0/4) | 0% (0/4) |  |
| *Saimiri sciureus* | Cebidae | 18 | 39% (7/18) | 0% (0/18) | 11.1% (2/18) |  |
| *Symphalangus syndactylus* | Hylobatidae | 2 | 50% (1/2) | 100% (2/2) | 0% (0/2) |  |
| *Theropithecus gelada* | Cercopithecidae | 1 | 100% (1/1) | 100% (1/1) | 0% (0/1) |  |
| *Varecia rubra* | Lemuridae | 11 | 55% (6/11) | 100% (11/11) | 9.1% (1/11) |  |
